# The grimace scale reliably assesses chronic pain in a rodent model of trigeminal neuropathic pain

**DOI:** 10.1016/j.ynpai.2017.10.001

**Published:** 2017-11-01

**Authors:** Titilola Akintola, Charles Raver, Paige Studlack, Olivia Uddin, Radi Masri, Asaf Keller

**Affiliations:** a Program in Toxicology, University of Maryland School of Medicine, Baltimore, MD, USA; b Program in Neuroscience, University of Maryland School of Medicine, Baltimore, MD, USA; c Department of Anatomy and Neurobiology, University of Maryland School of Medicine, Baltimore, MD, USA; d Department of Advanced Oral Sciences and Therapeutics, School of Dentistry, Baltimore, MD, USA

**Keywords:** Chronic pain, Constriction nerve injury, Behavior, Pain metrics

## Abstract

The limited success in translating basic science findings into effective pain management therapies reflects, in part, the difficulty in reliably assessing pain in experimental animals. This shortcoming is particularly acute in the field of chronic, ongoing pain. Quantitative analysis of facial expressions—the grimace score—was introduced as a promising tool, however, it is thought to reliably assess only pain of short or medium duration (minutes to hours). Here, we test the hypothesis that grimace scores are a reliable metric of ongoing neuropathic pain, by testing the prediction that chronic constriction injury of the infraorbital nerve (CCI-ION) will evoke significant increases in grimace scale scores. Mice and rats were subjected to CCI-ION, and tested for changes in mechanical hypersensitivity and in grimace scores, 10 or more days after surgery. Both rats and mice with CCIION had significantly higher grimace scores, and significantly lower thresholds for withdrawal from mechanical stimuli applied to the face, compared to sham-operated animals. Fentanyl reversed the changes in rat grimace scale scores, suggesting that these scores reflect pain perception. These findings validate the grimace scale as a reliable and sensitive metric for the assessment of ongoing pain in a rodent model of chronic, trigeminal neuropathic pain.

## Introduction

Despite advances in understanding the mechanisms of chronic pain, and despite significant commercial attempts to develop therapies for it, there has been limited progress in translating these investments to address the personal, societal and economic burden of chronic pain (Institute of Medicine, 2011). This failure has been a significant contributor also to the opioid epidemic (Skolnick and Volkow, 2016; Volkow and McLellan, 2016). The failure to translate research and industry investments in basic science into effective therapies is thought to reflect, at least in part, the difficulty in reliably assessing pain in experimental animals (Mogil, 2009). Particularly challenging is the ability to reliably quantify ongoing pain (Tappe-Theodor and Kuner, 2014), the major complaint of patients with chronic pain (Greenspan et al., 2004; Boivie, 2006; Bennett, 2012; Ekman and Rosenberg, 2005).

Several approaches have been developed to attempt to reliably monitor ongoing pain in experimental animal models (Mogil, 2009; Tappe-Theodor and Kuner, 2014; Mogil et al., 2010; Gregory et al., 2013; Burma et al., 2017; Munro et al., 2017). One of the most promising approaches relies on the analysis of facial expressions, an evolutionarily conserved ability to express emotions, including pain (Crook et al., 2014; Darwin, 1872; Williams, 2002). To adapt this approach to the study of ongoing pain, Mogil and collaborators have developed facial grimace scales for mice (Langford et al., 2010) and for rats (Sotocinal et al., 2011), and demonstrated that these objective metrics have a high accuracy and reliability for detecting ongoing pain. However, whereas Mogil et al reported that these scales are reliable for quantifying pain of moderate duration (from minutes to hours), they found that days and weeks after the induction of pain, animals displayed no distinct facial features (Langford et al., 2010; Sotocinal et al., 2011). This suggested that facial expressions cannot be used as a reliable metric of ongoing pain in neuropathic pain models, or in other models of chronic pain. This is because the transition from acute to ongoing pain in such models occurs 2 or more weeks after injury (Castro et al., 2017; Okubo et al., 2013; Masri et al., 2009).

Because of the acute need for reliable pain metrics in models of chronic pain, we reassessed the applicability of grimace score, in both rats and mice, for reliably detecting ongoing pain in neuropathic models. To test the hypothesis that grimace scores are reliable metric of ongoing neuropathic pain, we tested the prediction that chronic constriction injury of the infraorbital nerve—a procedure that results in profound hyperalgesia (Vos et al., 1994; Benoist et al., 1999; Okubo et al., 2013)—will evoke in experimental animals significant increases in grimace scale scores.

## Methods

We adhered to accepted standards for rigorous study design and reporting to maximize the reproducibility and translational potential of our findings as described in Landis et al. (2012) and in ARRIVE (Animal Research: Reporting In Vivo Experiments) Guidelines. Where appropriate, animals were randomly allocated to experimental or control groups, as described in Kim and Shin (2014). In all experiments the investigators were blinded to animal condition. A coded key of all specimens evaluated was kept and not shared with the investigators performing the experiments until data analyses were completed. We performed a power analysis to estimate the required samples needed for each experiment.

### Subjects

All procedures were approved by the University of Maryland, Institutional Animal Care and Use Committee, and adhered to National Research Council guidelines (Council, 2011). Male Sprague-Dawley rats (Envigo Laboratories, Frederick, MD) were 10–13 weeks old at the beginning of the study, and male C57BL/6 (The Jackson Lab, Bar Harbor, ME) were 10–12 weeks old. Rats were housed in pairs, and mice in cages of 4–6 individuals, all in limited-access animal rooms the animal facility. All animals were housed in polycarbonate cages at room temperature (23 ± 0.5 °C) on a 12 h light/dark cycle (lights on from 7:00 am to 7:00 pm), and allowed access to standard chow and drinking water ad-libitum throughout the study.

### Experimental design

Animals were handled and acclimatized to the experimenter and all apparatuses for 3 days before testing to reduce anxiety or stress. Handling and acclimatization involved daily, 5 min sessions whereby animals were gently held and stroked around the vibrissa pad area. Animals were then placed for 10 min in the facial grimace Plexiglass apparatus (8″ × 8″ inches for rats, 3″ × 3″ for mice) containing homecage bedding (for rats) or a bare floor (mice). Two days before the surgery, baseline facial von Frey and facial grimace scale readings were taken. Nerve constriction surgery was performed and the animals were allowed to recover for 5–7 days in their home cage, and were monitored daily. After the recovery period, von Frey thresholds and grimace scores were recorded again at either 10 days or 27 days post-injury. Mice were tested 21 to 24 days after injury.

### Chronic constriction injury of the infra-orbital nerve (CCI-ION)

We used a rodent model of neuropathic pain, evoked by chronic constriction of the infraorbital nerve (CCI-ION) (Benoist et al., 1999; Okubo et al., 2013; Castro et al., 2017). Animals were anesthetized with ketamine and xylazine, and intra-oral surgery was performed under aseptic conditions. An incision was made along the gingivobuccal margin, beginning distal to the first molar. The ION was freed from surrounding connective tissue, and loosely tied using silk thread (4–0), 1–2 mm from the nerve’s exit at the infraorbital foramen. We used silk thread, rather than chromic gut as originally described by Benoist et al (1999), because silk ligatures demonstrate more stable neuropathic pain behaviors in mouse CCI-ION models (van der Wal et al., 2015).

### Facial von Frey test

A series of calibrated von Frey filaments were applied to the orofacial skin, at the cutaneous site innervated by the ION. An active withdrawal of the head from the probing filament was defined as a response. We used the up-down method to determine withdrawal thresholds, as described previously (Chaplan et al., 1994).

### Facial grimace test

Animal were placed in a Plexiglas chamber, and video camera images (Canon) were recorded for 20 min. Scoring the facial expressions is a semi-automated procedure that uses the “face finder” application (Sotocinal et al., 2011)—generously provided to us by J.S. Mogil—to capture appropriate screen shots for scoring. The grimace scale quantifies changes in a number of “action units” including orbital tightening, nose-cheek bulge, whisker tightening and ear position for rats, and orbital tightening, nose bulge, cheek bulge, ear position, and whisker change for mice. Face images were screened, labeled, randomly scrambled and scored, with the experimenter blinded to the treatment groups (pre/post injury or drug-treated) and identity of each image. Ten screenshots were selected for each animal—per treatment condition or time-point—and on each image, each action unit was given a score of 0, 1, or 2, as previously described (Langford et al., 2010; Sotocinal et al., 2011). Mean grimace scores were calculated as the average score across all the action units.

### Drug administration

Fentanyl citrate (West-Ward Pharmaceuticals, Eatontown, NJ) was administered to a sub-group of rats (n = 14) one day after post-CCI baselines were recorded. The dose of 25 μg/kg was selected based on dose response studies performed in a separate group of rats (data not shown). Five minutes after injections, animals were tested for grimace scores, as above, and then for von Frey thresholds.

## Results

### CCI-ION produces mechanical sensitivity

**Fig. 1:**
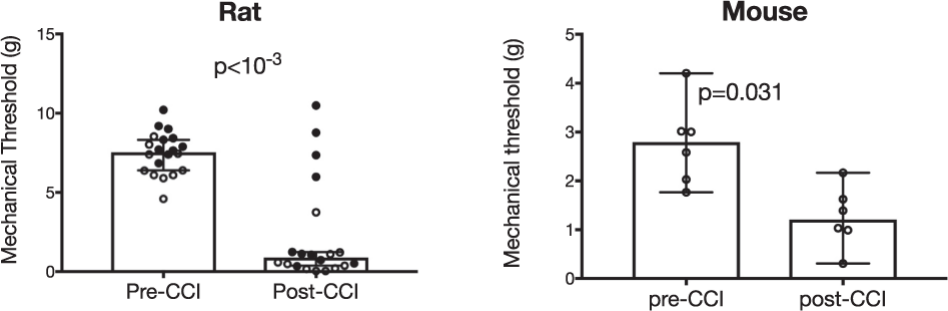
CCI-ION significantly reduces mechanical withdrawal thresholds, in both rats (left) and mice (right). For rats, filled circles are data collected 27 days after CCI, and open circles are data collected 10 days after CCI. Group data are shown as medians with 95% confidence intervals.

Chronic constriction injury of the infraorbital nerve (CCI-ION) results in significant mechanical hypersensitivity. Fig. 1 compares thresholds computed before and after CCI-ION in rats and mice. It depicts data from each animal, as well as medians and 95% confidence intervals (CI) of withdrawal thresholds from mechanical stimuli applied to the ipsilateral whisker pad. For rats, we include in the post-CCI group animals tested 10 days after CCI (open circles, n = 10) or 27 days after CCI (closed circles, n = 10). For the 10 day group, thresholds were reduced from 6.39 g (5.9 to 7.5, 95% Cl) to 0.43 g (0.002 to 1.6, 95% Cl), (p = .0020, Wilcoxon test). For the 27 day group, median thresholds were reduced from 8.11 g (7.6 to 9.0 g, 95% Cl) to 1.18 g (0.9 to 6.6, 95% Cl) (p = .0195, Wilcoxon test). Thresholds were significantly reduced in both groups, however, 5 of the 20 animals showed no significant change in their thresholds post-CCI. There were no significant differences in post-CCI thresholds between the 10 day and 27 day groups. We therefore combined the data from the two groups, as depicted in Fig. 1. For the group data, thresholds were reduced from 7.55 g (6.9 to 8.1, 95% Cl) to 0.90 g (0.8 to 3.8, 95% Cl), (p < 10^−3^, Wilcoxon test). In mice, thresholds were reduced from 2.80 g (1.9 to 3.7, % Cl) to 1.21 g (0.6 to 1.9, 95% Cl, (p = .031, Wilcoxon test). These findings confirm that CCI-ION results in significant mechanical hypersensitivity that appears as early as 10 days after CCI, and lasts at least 3 weeks, in both rats and mice.

### Facial grimace scale reliably reports ongoing pain

**Fig. 2:**
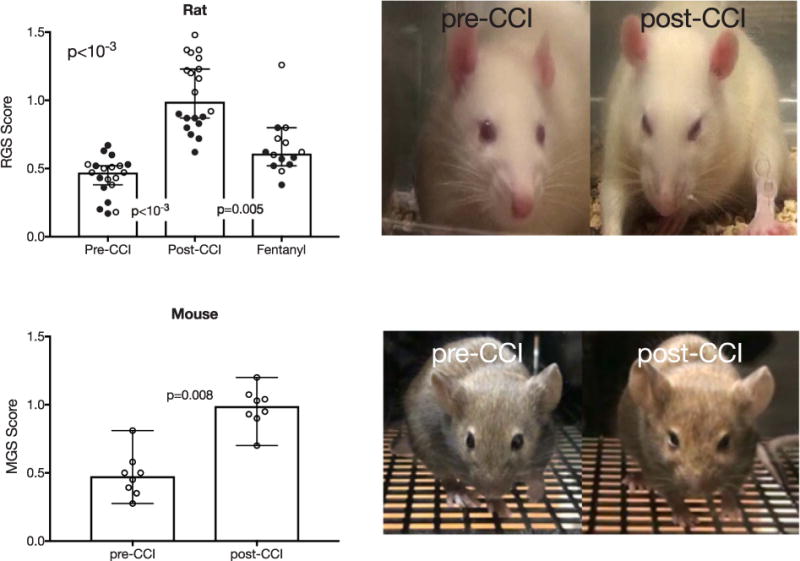
CCI-ION significantly increases grimace scale scores in both rats (above) and mice (below). Group data are shown as medians with 95% confidence intervals. For rats, filled circles are data collected 27 days after CCI, and open circles are data collected 10 days after CCI. Fentanyl administration, in rats, significantly reduced grimace scores. Sample images for each species are shown on the right.

To test the hypothesis that rats with mechanical hypersensitivity experience ongoing pain, we video-recorded spontaneous facial expressions and computed, from the same groups of animals, rat grimace scale (RGS) and mouse grimace scale scores (MGS) recorded from the facial grimace test (see section “Facial Grimace test”). Fig. 2 depicts data from each animal, as well as medians and 95% confidence intervals (CI) of RGS scores before and after CCI-ION. Again, we include animals tested 10 days after CCI (open circles, n = 10) or 27 days after CCI (filled circles, n = 10). For the 10 day group, RGS scores increased from 0.47 (0.4 to 0.5, 95% Cl) to 1.27 (1.2 to 1.4, 95% CI), (p = .002, Wilcoxon test). For the 27 day group, RGS scores increased from 0.48 (0.3 to 0.6, 95% Cl) to 0.85 (0.8 to 0.9, 95% Cl), (p = .002, Wilcoxon test). RGS scores were significantly increased in both groups and there were no significant differences in post-CCI scores between the 10 day or 27 day groups, therefore, we combined the data from the two groups. The group data (Fig. 2a) show RGS scores increased from 0.47 (0.4 to 0.5, 95% Cl) to 0.99 (0.9 to 1.2, 95% Cl,) (p < 10^−3^, Wilcoxon test).

We also tested the reliability of the grimace scale in mice (n = 8) using the mouse grimace scale (MGS; see section “Facial Grimace test”). Fig. 2b shows significantly increased MGS scores from 0.48 (0.3 to 0.6, 95% Cl) to 0.99 (0.9 to 1.1 95% Cl), (p = .0078, Wilcoxon test) after CCI. These findings confirm that rodents with mechanical hypersensitivity exhibit significant ongoing pain behaviors and confirm the efficacy of the facial grimace test.

### Facial grimace test is sensitive to analgesic-induced changes

To further confirm the reliability of the facial grimace scale, we assessed its sensitivity to changes in ongoing pain behavior. In CCI-ION injured animals, we tested the effect of Fentanyl, a fast-acting opioid, on RGS scores. Because of Fentanyl’s analgesic effects, we hypothesized that Fentanyl will reduce pain behavior resulting in lower RGS scores in comparison to post-CCI scores. To test this, a subset of rats (n = 14) were treated with 25 μg/kg of Fentanyl 1 day after Post-CCI data was recorded. Fig. 2a shows animals that received s.c. injections of Fentanyl 28 days after CCI (filled circles, n = 7) or 11 days after CCI (open circles, n = 7). Consistent with our prediction, Fentanyl significantly reduced post-CCI median RGS scores from 0.99 (0.9 to 1.2, 95% Cl) to 0.61 (0.5 to 0.8, 95% Cl), (p = .005, Kruskal Wallis test).

## Discussion

As described in the Introduction, research into the mechanisms of chronic pain, and studies of potential therapies for chronic pain, have been complicated by the absence of a consensus on reliable, objective and reproducible pain metrics (Mogil, 2009). This shortcoming is particularly acute in studies attempting to quantify ongoing, or “spontaneous” pain, and the affective aspects of pain (Bonasera et al., 2015; Craig, 2009; Mogil, 2009; Tappe-Theodor and Kuner, 2014). Mogil and collaborators addressed these shortcomings by developing the mouse grimace scale (Langford et al., 2010), and, later, the rat grimace scale (Sotocinal et al., 2011). They demonstrated that these scales provide accurate, standardized behavioral coding, with high accuracy and reliability, of ongoing pain. These rodent grimace scales confirmed, and quantified, the utility of facial expression as reliable metrics of pain in humans and other mammals (Darwin, 1872; Dalla Costa et al., 2014; Hampshire and Robertson, 2015; Guesgen et al., 2016; Viscardi et al., 2017).

In their original description of the rodent grimace scales, Mogil and collaborators reported that the these scales are reliable for assessing pain of short and moderate duration, but that lasting, chronic pain may not be reliably assessed with this tool (Langford et al., 2010; Sotocinal et al., 2011). Our aim was to reevaluate this by testing the hypothesis that facial expression reliably diagnoses ongoing pain in rodent models of chronic pain. We focused on a model of neuropathic pain induced by chronic constriction of the infraorbital nerve (CCI-ION) (Vos et al., 1994; Benoist et al., 1999), because it produces profound hyperalgesia. Further, we previously demonstrated that the transition from acute to lasting pain in this model occurs at 2 weeks after CCI-ION (Okubo et al., 2013). This allowed us to focus on a mechanistically-identifiable post-injury period that corresponds to chronic pain, to directly test our hypothesis.

Consistent with our hypothesis, we find that facial grimace scores are significantly increased, in both rats and mice, days as early as 10 days after CCI-ION, and that these increases are accompanied by trigeminal hyperalgesia. These changes were reversed by the administration of an opioid analgesic, suggesting that they reflect increased pain perception.

Although, when analyzed as a group, mechanical withdrawal thresholds were significantly and strikingly reduced after CCI-ION, in 5 of the rats thresholds appear to not have changed (Fig. 1a). This likely reflects unintentional, excessive nerve constriction in these animals, resulting in reduced sensitivity to evoked stimuli (Vos et al., 1994; Benoist et al., 1999; Kernisant et al., 2008). Interestingly, these 5 rats did display increased RGS scores, suggesting that they experience ongoing pain. Such dissociation between “reflexive” and “affective” pain metrics has been reported previously for several pain models (Urban et al., 2011; Rutten et al., 2011; Pratt et al., 2013; Boyce-Rustay et al., 2010; Barthas et al., 2015), highlighting the importance of assessing multiple pain metrics that reflect different pain dimensions (Melzack, 1999; Price, 2002).

We considered the possibility that CCI-ION surgery might have produced neuromuscular abnormalities that affected facial muscles, thereby affecting facial grimace. We consider this unlikely for several reasons. (1) The infraorbital nerve lies superficially in the oral cavity, such that little, if any, muscular damage is likely to occur during surgery, and branches of the facial nerve innervating muscles of facial expression are not in the vicinity of the incision; (2) The ION contains only sensory fibers, and no motor fibers that might be affected by the CCI-ION; (3) Fentanyl reversed the increased grimace scores, consistent with CCI-ION resulting in a pain-like condition, and not in neuromuscular abnormalities. If there were neuromuscular abnormalities, fentanyl is unlikely to have reversed them, as opioids produce muscular rigidity, and not relaxation, and this effect occurs only at high concentrations, and through central mechanisms (Christian et al., 1983; Desaiah and Ho, 1979; van den Hoogen and Colpaert, 1987). For these reasons we find it unlikely that CCI-ION resulted in neuromuscular deficits, suggesting that the resulting increases in grimace scores are related to the expression of ongoing pain.

Our findings are applicable to pain assays following CCI-ION in rats and mice, a procedure that produces severe pain (see section “Chronic constriction injury of the infra-orbital nerve (CCI-ION)” above). It is possible that models of neuropathic pain that affect other peripheral nerves, such as the sciatic nerve, produce less severe pain, and, therefore, do not manifest changes in grimace scores (Langford et al., 2010). This interpretation is consistent also with evidence suggesting that trigeminal and spinal pain may involve different mechanisms, perhaps involving different brain structures (Schmidt et al., 2015; Meier et al., 2014). Significant and lasting increases in grimace scale scores have also been reported recently in other chronic pain models, including rat models of spinal cord injury (Wu et al., 2016; Schneider et al., 2017), a mouse model of multiple sclerosis (Duffy et al., 2016), a rat model of migraine headaches (Harris et al., 2017), and a rat model of tooth movement (Gao et al., 2016). In contrast, rodent pain models that may produce more mild pain, such as those involving constriction or ligation of hindlimb nerves, may produce tactile hypersensitivity but only transient, or no increases in grimace scores (Langford et al., 2010; De Rantere et al., 2016; Kawano et al., 2014).

These findings suggest that grimace scores are reliable indicators of ongoing, chronic pain in both rats and mice with trigeminal neuropathic pain.
